# Sustainable Functionalization of PAN to Improve Tinctorial Capacity

**DOI:** 10.3390/polym13213665

**Published:** 2021-10-24

**Authors:** Vasilica Popescu, Ingrid Ioana Buciscanu, Melinda Pruneanu, Stelian Sergiu Maier, Angela Danila, Vasilica Maier, Marius Pîslaru, Vlad Rotaru, Irina Niculina Cristian, Andrei Popescu, Bogdan Istrate, Alexandra Cristina Blaga, Florin Ciolacu, Igor Cretescu, Petronela Chelariu, Marina Marin

**Affiliations:** 1 Department of Chemical Engineering in Textiles and Leather, Faculty of Industrial Design and Business Management, “Gheorghe Asachi” Technical University of Iasi, 700050 Iasi, Romania; ingrid-ioana.buciscanu@academic.tuiasi.ro (I.I.B.); melinda.pruneanu@academic.tuiasi.ro (M.P.); stelian-sergiu.maier@academic.tuiasi.ro (S.S.M.); angela.danila@academic.tuiasi.ro (A.D.); vasilica.maier@academic.tuiasi.ro (V.M.); rotaruvlad1980@gmail.com (V.R.); petronelachelariu@yahoo.com (P.C.); marinamarin9817@gmail.com (M.M.); 2 Department of Engineering and Management, Faculty of Industrial Design and Business Management, “Gheorghe Asachi” Technical University of Iasi, 700050 Iasi, Romania; marius.pislaru@academic.tuiasi.ro; 3 Design and Engineering of Textile Products, Faculty of Industrial Design and Business Management, “Gheorghe Asachi” Technical University of Iasi, 700050 Iasi, Romania; irina-niculina.cristian@academic.tuiasi.ro; 4 Department of Machine Design, Mechatronics and Robotics, Faculty of Mechanical Engineering, “Gheorghe Asachi” Technical University of Iasi, 700050 Iasi, Romania; andrei.popescu@academic.tuiasi.ro (A.P.); bogdan.istrate@academic.tuiasi.ro (B.I.); 5 Department of Organic, Biochemical and Food Engineering, “Cristofor Simionescu” Faculty of Chemical Engineering and Environmental Protection, “Gheorghe Asachi” Technical University of Iasi, 700050 Iasi, Romania; alexandra-cristina.blaga@academic.tuiasi.ro; 6 Department of Natural and Synthetic Polymers, “Cristofor Simionescu” Faculty of Chemical Engineering and Environmental Protection, “Gheorghe Asachi” Technical University of Iasi, 700050 Iasi, Romania; florin.ciolacu@academic.tuiasi.ro; 7 Department of Environmental Engineering and Management, “Cristofor Simionescu” Faculty of Chemical Engineering and Environmental Protection, “Gheorghe Asachi” Technical University of Iasi, 700050 Iasi, Romania; icre@ch.tuiasi.ro

**Keywords:** functionalization, polyacrilonitrile, iodinehydrin, iodine-oxime, nitrile oxides, coloring

## Abstract

This study may open a new way to obtain the coloration of a polymer during functionalization. Two polyacrylonitrile (PAN) polymers in the form of textile fibers (*Melana* and *Dralon L*) were subjected to functionalization treatments in order to improve the dyeing capacity. The functionalizations determined by an organo-hypervalent iodine reagent developed in situ led to fiber coloration without using dyes. KIO_3_ was formed in situ from the interaction of aqueous solutions of 3–9% KOH with 3–9% I_2_, at 120 °C. The yellow-orange coloration appeared as a result of the transformations in the chemical structure of each functionalized polymer, with the formation of iodinehydrin groups. The degree of functionalization directly influenced the obtained color. The results of the Fourier Transform Infrared Spectroscopy (FTIR), Scanning Electron Microscopy (SEM), Energy Dispersive X-ray Spectroscopy (EDX), Map and Temogravimetric Analysis (TG) plus Differential Thermal (DTA) analyses indicated the presence of new functional groups, such as iodine-oxime. The X-ray diffraction (XRD) analysis confirmed the change of the crystalline/amorphous ratio in favor of the former. The new groups introduced by functionalization make it possible to dye with classes of dyes specific to these groups, but not specific to PAN fibers, thus improving their dyeing capacity.

## 1. Introduction

The functionalization of acrylic polymers is performed in order to enhance their reactivity and improve functionality, with impact on aesthetical and comfort properties. The color and appearance of PAN fibers depend on their tinctorial properties, which are generally satisfactory, but the dyeing process is complex and uneven shades are a common defect in PAN fiber dyeing. The lighter the desired color, the bigger the staining risk. Anionic leveling or cationic retarders are used to surpass this inconvenience but their use in dyeing recipes make the process more complicated and polluting, which is why they must be used in strictly controlled amounts.

The amount of retarder must be rigorously calculated according to a well-determined formula that involves knowing the specific indices of the cationic dyes and the acrylic polymer.

Reactive and functional PAN fibers were obtained by chemical changes of the polymer structure. Reported functionalization agents were: alkalis [1], amines such as dimethylaminopropylamine [2], ethylenediamine [3], hydrazine [4], urea or hydroxylamine [5,6,7], and primary or secondary acyclic aliphatic amines, diethylamine and diethylenetriamine [8]. Our previous work reports PAN functionalization with alkalis, amines and eco-friendly agents such as chitosan and monochlorotriazinyl-β-cyclodextrin (MCT β-CD) [9,10,11,12,13].

Chemical transformations of the acrylic polymer/copolymer consist of:

- Conversion of the ester groups of vinyl acetate (VA) into hydroxyl groups; and
- Conversion of nitrile groups into other different functional groups (oxime, hydroxamic acid, mono/disubstituted amidines), depending on the basicity of the functionalization agent [9,10,11,12,13].

The chemical reactions types involved in PAN functionalization are saponification, hydrolysis, amination and N-acylation. Generated functional groups can act as grafting sites for eco-friendly compounds [9,10,11,12,13].

The novelty of this study consists in the enhancement of the tinctorial capacity of PAN fibers through a sustainable dye-free functionalization. The coloration effect is the result of certain chemical modifications of the acrylic polymer, produced by functionalization.

Specialty literature shows that PAN changes color only when it is subjected to thermal treatment at above 240 °C during stabilization, as the first stage of PAN conversion into carbon fiber [14]. Color appears as result of the formation of the polyimine cycle in nitrogen [15], oxygen [16,17,18,19] or air [20], at high temperature. Stabilization in a mixture of air and ammonia, NH_3_ at 260 °C turns PAN’s color from white to yellow and finally to black, depending on treatment severity [21].

Chemical and physical changes in the PAN structure are insignificant if the treatment is performed at temperatures below 140 °C. Above this temperature, oxygen in the air can sensitize the nitrile group (polyimine cycles occur) and cause crosslinking between the macromolecular chains of PAN [19,22].

Nonetheless, an adequate oxidizing agent can produce PAN coloration even at temperatures below 140 °C. For example, potassium permanganate, KMnO_4_ acts as a catalyst for the cyclization of nitrile groups at 80 °C, when color appears in the PAN fiber [23,24,25].

In the present work, PAN coloration is due to functionalization with alcoholic solutions of I_2_/KI in alkaline medium. An iodine-potassium iodide solution alone does not stain PAN materials, but produces swelling [26,27], change of electrical conductivity and dielectric constant [26,27,28,29] and alteration of the internal structure reflected in the decrease of the crystalline/amorphous ratio [27]. These effects are related to doping, which occurs in the chemical treatment of PAN, irrespective of the iodine states of (1) vapors [28], (2) crystals dissolved in ethanol/water solution [30], or (3) an aqueous I_2_/KI solution in which the triiodide ion, I_3_^−^ is generated [31,32].

The iodine-potassium iodide solution determines PAN doping and swelling. Cross-linking in swelled PAN is temperature dependent, i.e., it decreases with temperature rise [14,33].

In order to avoid doping and promote PAN functionalization, we studied PAN treatment with I_2_/KI solution in the presence of potassium hydroxide, KOH. It is known that KOH can act as a catalyst in chemical reactions taking place at high temperature [34].

A step by step technique was applied to elucidate the mechanism of chemical transformations of PAN treated with I_2_/KI in KOH solution, examining: (1) PAN functionalization with potassium hydroxide, KOH; (2) PAN functionalization with I_2_/KI solutions; and (3) functionalization with I_2_/KI in KOH solution. For each working variant, the tinctorial properties of the acrylonitrile polymer as a function of the treatment solution composition were assessed. FTIR, EDX and XRD analyses confirmed the presence of newly formed functional groups. Treatment with KOH + I_2_/KI for 60 min at 120 °C determined PAN functionalization through the formation of iodine-oxime groups, which impart a yellow-orange color to the PAN fiber. The essential role in functionalization is played by potassium iodate, KIO_3_ which is generated in situ and releases molecular oxygen, O_2_ at 120 °C. Oxygen reacts mainly with the nitrile groups and generates oxidized unstable intermediates that readily react with iodine to form cyanohydrins of the iodine-oxime type. These are considered as pre-stabilization stages, which take place under physical (temperature 120 °C, pressure > 1 atm, aqueous medium) or chemical (KIO_3_/O_2_ generated in situ) stimuli. Pre-stabilization generates in the increase of optical density and shrinkage of the treated fabric.

The novelty of this study consists in the sustainable functionalization of PAN fibers, which results in a deep and uniform yellow-orange coloration in the absence of any dyestuff. The proposed process reduces pollution and water/dye/chemical/energy demands, as compared with the conventional PAN dyeing process with cationic dyes. The proposed functionalization improves the tinctorial capacity of PAN fibers in terms of broadening the range of dyes usable for the polyacrylonitrile fibers. Thus, functionalized PAN gains an affinity for anionic dyes by means of the newly formed functional groups, which are able to establish ionic bonds with this class of dyes that is non-typical for PAN.

It is the aim of this paper to carry out PAN fiber coloration through functionalization by oxidative reactions in the presence of an environmentally sustainable organo-hypervalent iodine reagent.

## 2. Materials and Methods

### 2.1. Materials

Acrylic materials used in this study were 1 × 1 rib knit fabrics, in two vertical gauge values: 9.0 (sample V1) and 10 (sample V2). The commercial acrylic fibers used for the tested knits were: Melana (Rifil S.A., Savinesti, Romania) and Dralon L (Dralon GmbH Werk Ling, Lingen, Germany).

The samples were knitted on a E12 gauge (12 needles per inch) Stoll CMS-500 flat knitting machine (Karl Mayer STOLL Textilmaschinenfabrik GmbH, Reutlingen, Germany). Gauge value has an influence upon the fabric thickness in the vertical direction. The sample with gauge 9 (variant V1) has a higher vertical gauge than the sample with gauge 10 (variant V2), where the vertical gauge is the number of knitted rows per inch.

**Figure 1:**
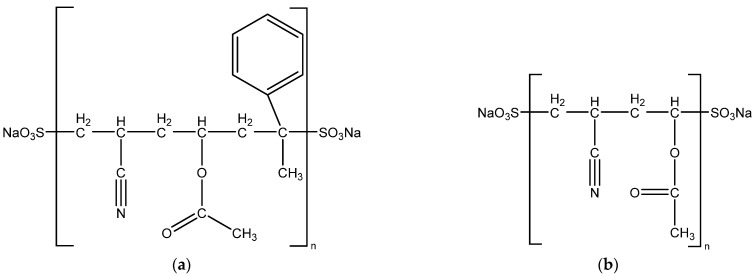
Chemical structures of the investigated acrylic polymers: (**a**) “Melana”; (**b**) “Dralon L”.

Chemical structures of the acrylic polymers from which the fibers are made are given in Figure 1.

Other names for Dralon L: vinyl acetate/acrylonitrile copolymer and vinyl acetate/acrylonitrile polymer.

Other names for Melana: acrylonitrile/vinyl acetate/alpha methylstyrene copolymer and acrylonitrile/vinyl acetate/alpha methylstyrene polymer. The Romanian brand Melana refers to a ternary polymer (acrylonitrile 85%, vinyl acetate 10% and alpha-methylstyrene 5%) acrylic fiber obtained through radical polymerization with the redox system potassium persulfate—sodium metabisulfite.

### 2.2. Reagents and Chemicals

Potassium hydroxide (KOH), acetic acid (CH_3_COOH), ethanol (C_2_H_5_OH), and iodine-potassium iodide I_2_/KI solution were purchased from Carl Roth GmbH (Karlsruhe, Germany).

The alkaline pH required for functionalization was provided by KOH (pK_b_ = 0.5), as a weaker base than NaOH (pK_b_ = 0.2) [35]. All chemicals were of reagent grade.

### 2.3. Functionalization Experiments

The acrylic fibers Melana and Dralon L were subjected to chemical treatments and the functionalization effects were assessed. The functionalization experiments were conducted with I_2_/KI solutions in the presence or absence of an alkali, namely KOH, in accordance with the experimental protocol given in Table 1.

Functionalization treatments were conducted in sealed stainless-steel vessels at two different temperatures: room temperature (20 °C) and 120 °C, respectively. Reagent amounts were calculated as percent on weight of fabric samples, in the range 3–9%; the liquor ratio (M) was 1:15.

Functionalization treatments were followed by rinsing with warm and cold water, neutralization with acetic acid, wringing and air drying.

### 2.4. Analysis of the Functionalized Fibers

#### 2.4.1. FTIR Analysis

The FTIR spectra of the acrylic materials were recorded on a Bruker Optics equipment(Bruker Optik GmbH, Ettlingen, Germany), comprising a TENSOR 27 FTIR spectrophotometer(Bruker Optik GmbH, Ettlingen, Germany), adequate mainly for near-IR, coupled with a HYPERION 1000 microscope equipped with a standard 15× objective. The standard DLaTGS detector works in the 7500–370 cm^−1^ spectral range, with a resolution of 4 cm^−1^. The TENSOR 27 spectrophotometer is equipped with a He–Ne laser that operates at a wavelength of 633 nm and an output power of 1 mW, and presents a ROCKSOLID alignment of the interferometer. TENSOR 27 was assisted by OPUS software, which allowed for the acquisition of interactive video data.

The liquid nitrogen-cooled MCT detector covered the spectral range 600–7500 cm^−1^; measured aria was optimized to 250 μm, but can reach a minimum of 20 μm.

#### 2.4.2. XRD Analysis

XRD analysis was performed on an X’PERT PRO MRD X-ray generator (PANalytical, Almelo, The Netherlands) with the following characteristics: tube voltage = 35 kV, tube current = 20 mA., vacuum pressure = (1/2) mbar, slit width = 80 μm, counter slit width = 250 μm, wavelength of Cu kα radiation λ = 1.5418 A^0^, the counter- sample distance A = 20 cm, capillary diameter (d) = 1 mm, working temperature 22.5 ± 0.5 °C. Monochromatic Cu kα (λ = 1.54 A^0^) radiation was obtained with a nickel filter of 10 μm thickness, used to irradiate acrylic fibers packed in a Mark capillary tube(Capillary Tube Supplies Ltd., Rose Cottage, United Kingdom) of 1 mm diameter.

#### 2.4.3. SEM + Map + EDX

The SEM plus Map plus EDX analyses were performed on an SEM microscope model VEGA II LSH (TESCAN S.R.O., Brno, Czech Republic) coupled with a 3rd generation EDX detector, model QUANTAX QX2 (BRUKER Optics, Ettlingen, Germany).

The main features of the microscope were a tungsten heated cathode, resolution 3 nm at 30 kV, scanning speed from 200 ns to 10 ms per pixel, magnification 13–1.000.000× in resolution mode at 30 kV, accelerating voltage 200 V to 30 kV and working pressure below 1 × 10^−2^ Pa. The XFlash EDX detector(Bruker Optik GmbH, Ettlingen, Germany), used for qualitative and quantitative analysis is 10 times faster than conventional Si (Li) detectors.

The SEM-EDX coupling allows, at the same time, microphotogram acquisition, surface imaging with atom mapping, and determination of the elemental composition (in mass or molar fractions) of a microstructure or a selected area of a sample.

#### 2.4.4. Thermal Resistance/Thermal Conductivity

Thermal resistances of the acrylic fabrics was determined on a Permetest Sensora device(Sensora Instruments & Consulting, Liberec, Czech Republic).

#### 2.4.5. TG and DTA Thermal Analysis/Thermogravimetry

Thermogravimetric analysis was performed on a computer-aided Linseis STA PT-1600 (Linseis Messgeraete GmbH, Selb, Germany) thermobalance with simultaneous recording of the thermogravimetric curves. The working conditions were heating rate 10 °C/min in a dynamic air atmosphere and gas flow of 50 mL/min, maximum temperature 800 °C, samples weighed 50 mg, as measured on an electronic balance model PARTNER AS220/C/2.

#### 2.4.6. Color Measurements

Colorimetric measurements CIELab and color intensity (K/S), were performed on functionalized samples, and on functionalized and dyed samples. To prove the presence of functional groups in the polymer chain, dyeing was performed with non-typical dyes, such as acid dyes.

Color was quantified based on the CIELab color model, using the experimental determination of L *, a *, b *, C * and h * values on a Datacolor Sprectroflash SF300 spectrophotometer(Datacolor, Lucerne, Switzerland). The significance of coloristic measurements are as follows: L * stands for luminosity; a * and b * are the position of color on the red-green and yellow-blue coordinates; C * is color saturation and h * is color shade.

Color intensity of dyed fabric samples, K/S was calculated with the Kubelka–Munk equation:

K/S = [(1 − R)^2^]/2R (1)

where the reflectance of the dyed fabrics, R [%] was measured on the same Datacolor Sprectroflash SF300 spectrophotometer (Datacolor, Lucerne, Switzerland).

#### 2.4.7. Statistical Analysis

Experimental values of electrical conductivity and color intensity were subjected to statistical processing. Error of the mean, standard deviation (SD) and the coefficient of variation (CV) were calculated in Matlab and indicated on the related figures.

## 3. Results and Discussion

### 3.1. Mechanism of PAN Functionalization

The attempt to elucidate the mechanism of acrylic polymer functionalization was based on a thorough documentation of previous work regarding the behavior of acrylic polymers in alkaline media and their interaction with iodine or I_2_/KI solutions.

Treating PAN fibers with 2.5% NaOH aqueous solution at 100 °C produces functionalization due to the generation of amide or carboxyl functional groups, with no color change [9,10,11]. The development of a yellow-orange color was noticed when functionalization was carried out with amine compounds such as hydroxylamines [9], which converted the nitrile groups into imine groups, more precisely into oxime groups (HO-NH-C=N) [13].

Yue et al. [34] studied the effect of alkalis upon the nitrile group in acrylonitrile and concluded that alkaline compounds act as catalysts for the conversion of acrylonitrile into acrylic acid by hydrothermal reaction (300 °C) and that potassium hydroxide, KOH is the most effective alkaline agent. Studies on the interaction between PAN and iodine from alcoholic solutions of I_2_/KI put in evidence polyacrylonitrile doping, which is favored by temperature decrease [26,27,28,29,36]. Other authors [37,38] state that iodine penetrates the polymer crystalline zones and forms a complex with the polyacrylic chain due to its ability to interact with the nitrile group.

In fact, weak physical electrostatic interaction between the K^+^ ion and C=O/C≡N is more likely. The newly formed structure has a higher ionic conductivity, which is dependent on temperature and KI concentration [38].

Other studies have shown that the amorphous region is the host of polymer matrix doping with iodine salt [30,38], but iodine is easily washed off with water or acetone. Doping determines alteration of the crystalline:amorphous regions ratio, polymer swelling [26,27], and increase of electrical conductivity [28,30,36,37,38].

In alcoholic solutions of I_2_/KI, iodine is present as a triiodide ion, I_3_^−^. The presence of triiodide in the PAN matrix can be easily detected by the starch test, when the appearance of a blue-black color proves formation of the iodine-starch complex. When only iodide is present in the polymer, no color change will be noticed, because iodide does not react with starch [31,32].

In this study, the two acrylonitrile polymers acquired new functional groups consequent to treatment with potassium hydroxide solutions, in the presence or absence of alcoholic I_2_/KI solutions. When treatment was performed with KOH alone, two kinds of functional groups are generated:

1. Hydroxyl groups, -OH due to the saponification of acetate groups of vinyl acetate (VA), simultaneously with acetic acid, CH_3_COOH release; and
2. Carboxyl groups resulting from the hydrolysis of nitrile groups, via hydroxamic acid/amide as intermediates.

Our previous works have shown that PAN fiber functionalization with sodium hydroxide did not alter fiber color and generated acidic functional groups by fast hydrolysis because NaOH is a strong base (pk_b_ = 0.2). In the present work, alkaline pH is provided by KOH, a weaker base (pk_b_ = 0.5), the hydrolysis reaction rate is lower, and the reaction mechanism involves the formation of hydroxamic acid instead of amide as an intermediate product. The final product is the acidic group (-COOH) together with hydroxylamine, and NH_2_-OH as secondary product. Hydroxylamine has affinity for nitrile groups (not involved in functionalization) and interacts with these groups to generate oxime/amidoxime groups [39]. Possible reactions are as follows:

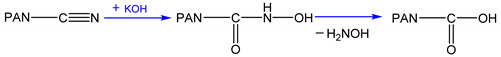
(2)

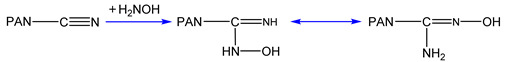
(3)

When PAN is treated with KOH and iodine in ethanol solution at 120 °C, potassium iodate and KIO_3_ generated in situ due to high temperature decompose and release molecular oxygen, O_2_ (Equations (5) and (6)); both KIO_3_ and O_2_ can oxidise the oxime/amidoxime/nitrile groups to nitrile oxide [40].

Generally, oxidation is accompanied by color change of PAN fibers, which is attributed to the pre-oxidation of the nitrile groups of the polymer. The pre-oxidation degree can be assessed by means of the extent of oxidation reaction (EOR) index, given by the formula: EOR = [I_1600_/(I_CN_ + I_1600_), where I_1600_ stands for the absorbance intensity from 1600 cm^−1^ and I_CN_ stands for the absorbance intensity of CN groups, in the IR domain. Extreme values of EOR are 0 and 1: when EOR = 0 none of the nitrile groups are pre-oxidised, and when EOR = 1, all nitrile groups in the polymer underwent oxidation [41].

Besides the pre-oxidation effect, when temperature exceeds 140 °C, oxygen may play an essential role in the cyclization process by two opposite effects: initiation of active site formation that is responsible for cyclization, and cyclization inhibition by rise of activation energy. When working temperature was lower than 140 °C, FTIR analysis of the treated acrylic polymer did not confirm the presence of cycles [42].

The hypervalent potassium iodate, KIO_3_ is a versatile and environmentally friendly reagent that can be used in different oxidative transformations [40].

Potassium iodate generated in situ is a hypervalent I(v) compound with high oxidizing strength and ability to convert amidoxime into nitril oxide, a nonisolable compound. Nitrile oxides are efficient 1,3-dipolar reactants in intra- or intermolecular cycloadditions [40,42]. Nitrile oxides are unstable and extremely reactive; they dimerize to form furoxans, hydrolyze to form N-hydroxyamides (hydroxamic acid) as final products, or interact with iodine in the reaction mixture. These functionalization reactions determine the permanent coloration of acrylic fibers in yellow-orange shades [40].

Chemical reactions involved in the conversion of nitrile groups of PAN chains in the above-mentioned functional groups are as follows:

(a) Conversion to amidoxime groups placed on the polymeric chain (Equations (2) and (3)), and in situ obtaining of potassium iodate at 120 °C (Equations (4)–(8)):

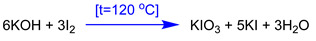

(4)



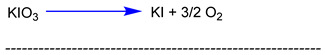

(5)



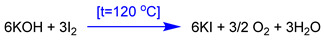

(6)



In reaction (4), I_2_ is both an oxidizing and a reducing agent (disproportionation/dismutation of elemental iodine), as follows:

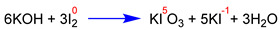


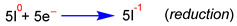
(7)

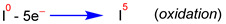
(8)

(b) Generation of nitrile oxides, through amidoxime oxidation by KIO_3_/O_2_ [43,44,45] (Equation (9)):

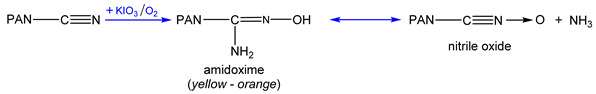

(9)



Oxidized nitrile groups are unstable intermediates (Equation (10)) [46,47,48] and readily turn into ionic forms that interact both with the I^−^ ion (derived from KI dissociation) and the H^+^ ion present in the reaction medium to generate polyacrylonitrile functionalized with iodine-oxime groups, PAN-C(I)=N-OH (Equation (11).




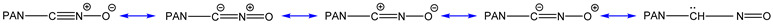

(10)



Nitrile oxides are mild oxidizing agents that release iodine from an acidified solution of KI [47]. The presence of both I^−^ and H^+^ ions makes possible the emergence of cyanohydrin of the iodinehydrin type [47]. Iodine present in iodinehydrin hinders dimerization and cyclization, having a stabilization effect [47].




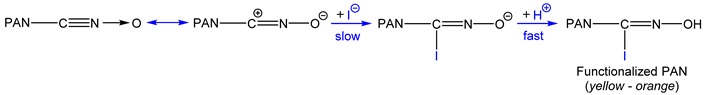

(11)



The pH measurement of treating solutions before and after functionalization showed pH decreased during treatment (Table 2), which proved the generation of acidic compounds during the reaction. Hydrogen iodide, HI, resulted from the reaction between water and KI, and acetic acid, CH_3_COOH, derived from the saponification of the acetate group present in vinyl acetate.

The iodine ion, I^−^ originates from the dissociation of KI, confirmed by solution conductivity before and after functionalization, increased the conductibility of the residual liquor after functionalization with KOH + I_2_ due to the increase of the K^+^ concentration, which demonstrates that potassium iodide is not involved in the PAN/KI complex formation [30]. According to some authors, formation of this complex indicates PAN doping with iodine [30].

The triiodide ion, I_3_^−^ is present in aqueous solutions of I_2_/KI, but is not detected when the starch test is applied on the functionalized acrylic polymers, and the characteristic blue-black coloration indicating iodine presence does not appear [31,32].

PAN functionalization was confirmed both by the FTIR spectra and the colorimetric measurements performed on treated yellow-orange samples.

### 3.2. FTIR Results for the Polyacrylonitrile Materials

**Figure 2:**
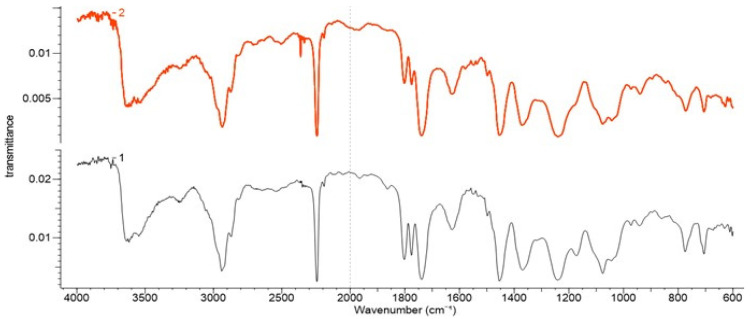
FTIR spectra of Melana: (**1**) pristine/untreated and (**2**) after functionalization.

**Figure 3:**
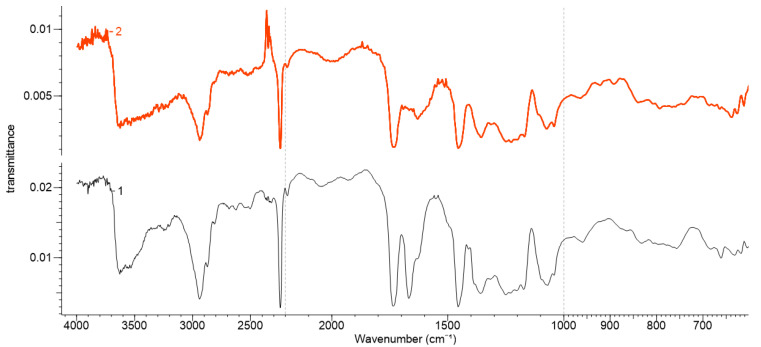
FTIR spectra of Dralon L: pristine (**1**) and (**2**) after functionalization.

Comparative examination of FTIR spectra of Melana and Dralon L before and after treatment confirmed functionalization, by the presence of carboxyl, of the oxime and hydroxyl groups in the treated samples (Figure 2 and Figure 3). These functional groups emerged as a result of chemical modifications of the nitrile group of the AN monomer and the acetate groups of the VA monomer, respectively. The nitrile group has an intense adsorption band at 2242 cm^−1^, while the aliphatic nitrile oxide intermediates have two adsorption bands at around 2330 cm^−1^ (C=N stretching) and around 1370 cm^−1^(-N=O stretching). The C=N stretching band is preferred for the identification of monomeric nitrile oxides [47].

Carboxyl groups (-COOH) generated by the alkaline hydrolysis of a part of the nitrile groups ere confirmed by the increase of 1746–1735 cm^−1^ (C=O stretching) and 1249–1240 cm^−1^ (C-O stretching) peak intensities. Newly formed carboxyl groups increase the oxygen content of the functionalized acrylic polymers, even if the saponification of ester groups of VA with formation of hydroxyl groups (-OH) is prone to deprive the polymer of oxygen.

The presence of oxime groups (C=N-OH) in Melana functionalized with iodine-oxime groups (C(I)=N-OH) as confirmed by the three characteristic IR bands: 3639 cm^−1^ (O−H), 1628 cm^−1^ (C=N) and 939 cm^−1^ (N−O). The peak at 628 cm^−1^ was assigned to iodine from the iodinehydrin group. Adsorbtion bands at 3539 cm^−1^ (O−H) and 1076 cm^−1^ (C–O stretch (s)) were assigned to hydroxyl groups derived from saponification of acetate groups of VA.

Dralon L functionalization with KOH + I_2_/KI was proven by the presence of adsorption bands characteristic to the newly formed functional groups, namely iodine-oxime/iodinehydrin, (C(I)=N-OH), and hydroxyl (-OH). New adsorption bands in the functionalized Dralon L were compared with the untreated material and assigned to oximes (3633 cm^−1^ (O−H), 1631 cm^−1^ (C=N) and 930 cm^−1^ (N−O)). The adsorption band at 638 cm^−1^ was attributed to iodine in Dralon L functionalized with iodine-oxime groups. The hydroxyl groups generated by the saponification of ester groups of VA had adsorption bands at 3537 cm^−1^ (O−H) and 1074–1070 cm^−1^ (C–O stretch (s)).

The absorption bands of existing functional groups in untreated PAN fibers [9,10,11,12] and those of newly born functional groups due to functionalization with KIO_3_ [38] are given in Table 3.

The adsorption band at about 762 cm^−1^ was assigned to potassium iodate, KIO_3_, while spectra modification due to functionalization could be observed at 761 cm^−1^ in Dralon L and at 773 cm^−1^ in Melana (Figure 2 and Figure 3).

According to the literature, KI determines a frequency shift of the C–H rocking vibration [38]. Such vibrational shifts were detected in the studied PAN polymers: from 839 cm^−1^ (Dralon L) and 863 cm^−1^ (Melana), to 841 cm^−1^ and 901 cm^−1^, respectively.

The comparative assessment of FTIR spectra of pristine and functionalized PAN fibers put in evidence:

- The decrease in the peak intensity of CH_3_ (C-H stretching), due to splitting of acetate group of VA and formation of OH group; and
- The decrease in the peak intensity of nitrile group (CN stretching), due to conversion into carboxyl and oxime groups.

The extent of oxidation reaction, EOR was calculated based on:

1. The intensity of peaks at around 1600 cm^−1^, associated with C=N stretching (namely 1625 cm^−1^ in Melana or 1629 cm^−1^ in Dralon L); and
2. The intensity of peaks associated with the nitrile groups.

Values of EOR after the KOH treatment were 0.4275 for Melana and 0.4832 for Dralon L. Fabrics functionalized with KOH + I_2_/KI had very closed values of EOR: 0.425 for Melana and 0.455 for Dralon L. The EOR values indicated the contribution of the oxidation reaction in the functionalization of each acrylic polymer studied herein.

### 3.3. XRD Results Interpretation

Literature data state that treating PAN with I_2_/KI results in a decrease of the polymer’s degree of crystallinity. The presence of KI in the polymer lowers the transition temperature, T_g_ from 90 °C to 71 °C and the melting point from 300 °C to 256 °C. The drop in the T_g_ value indicates an increase of the extent of the amorphous regions of the polymer [38].

Treating acrylic polymers with I_2_/KI in the presence of KIO_3_ (and O_2_) generated in situ results in the sensitization of nitrile groups by oxidation and the increase of crystallinity.

**Figure 4:**
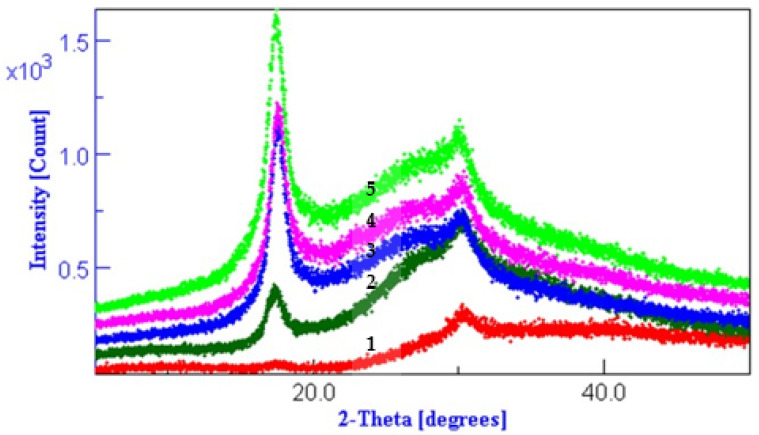
Diffractograms of Dralon L treated at 120 °C for 60 min: (**1**) control sample, (**2**) boiled in water, (**3**) 6% KOH, (**4**) 6% KOH plus 6% I_2_/KI, and (**5**) 6% KOH plus 6 % iodine.

**Figure 5:**
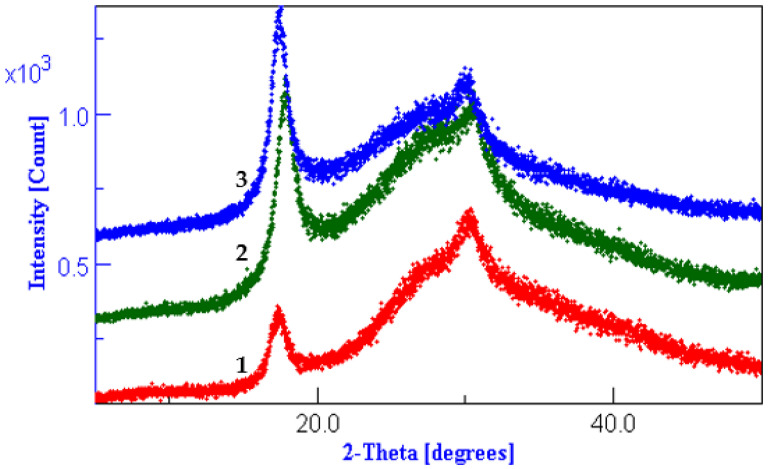
Diffractograms of Dralon L treated at 120 °C for 30 min with excess of reagents: (**1**) control sample, (**2**) sample V1 with KOH plus iodine, and (**3**) sample V2 with KOH plus iodine.

**Figure 6:**
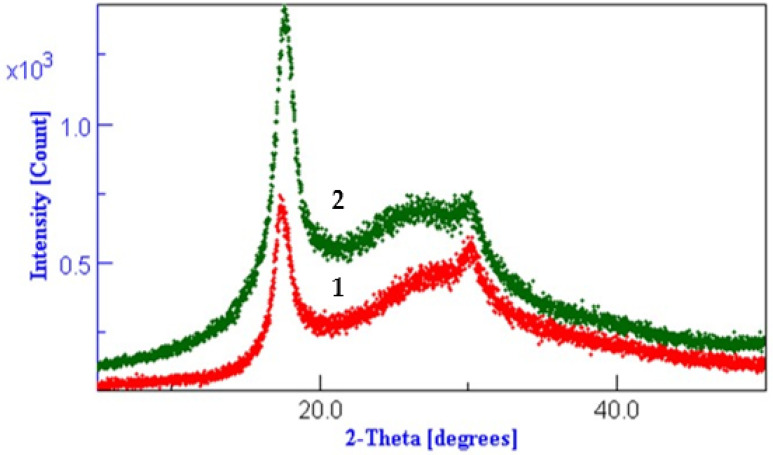
Diffractograms of Melana treated at 120 °C for 30 min with excess of reagents: (**1**) control sample, and (**2**) sample V1 with KOH plus iodine.

Melana and Dralon L are semi-crystalline acrylic polymers, which means that both amorphous and crystalline regions are present in the polymer chain (Figure 4, Figure 5 and Figure 6). XRD patterns of these polymers were extensively studied in our previous work [49].

Hydrothermal or chemical treatment in aqueous media determine the increase of Melana and Dralon L polymer crystallinity (Figure 4, Figure 5 and Figure 6). This behavior is in accordance with literature data, which indicate an increase of the polymer internal order when the working temperature is higher than 100 °C, such in case of microwave-assisted [49] or thermal treatment [50,51,52].

The working temperature of 120 °C was responsible for a certain degree of pre-fixation of acrylonitrile polymers, confirmed by the increase of peaks from positions 17 and 29 (2-Theta).

Lack of swelling and disturbance of the PAN fiber internal structure proves that iodine doping did not occur. Iodine attaches to the C atom of the oxime group to form the iodine-oxime/iodinehydrin bond, as proven by the FTIR analysis.

### 3.4. SEM Plus Map Plus EDX Results

**Figure 7:**
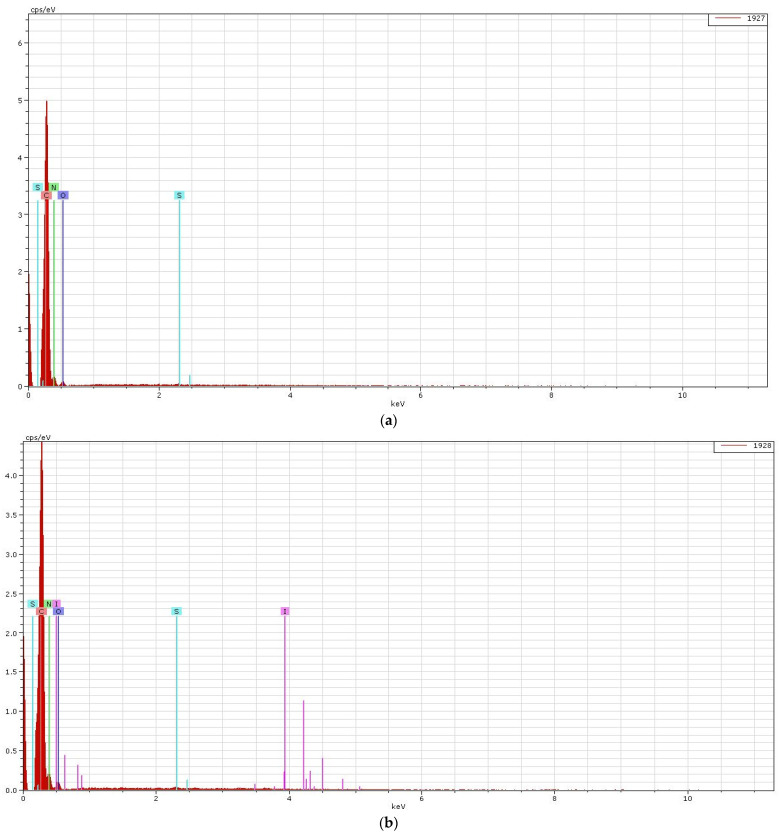
EDX spectrum of element analysis for Melana fibers: (**a**) untreated, and (**b**) after functionalization.

Results of quantitative and qualitative analyses provided by EDX and SEM will be discussed below. The results of the EDX quantitative elemental analysis of untreated and functionalized Melana are presented in Table 4 and Figure 7. The weight percent (wt.%), weight percentage (norm. wt.%) and atomic percentage (norm. at.%) of the elements present in the sample were calculated.

Quantitative analysis indicates the increase of oxygen (O_2_) content, which was due to generation of new -COOH functional groups. Carbon content decreased as a result of the ester group of VA splitting and the elimination of acetic acid, CH_3_COOH. The presence of iodine in functionalized Melana (Table 4) proved the formation of the iodine-oxime group (I-C=N-OH) and the persistence of iodine in the acrylic polymer, even after post-functionalization washing of samples. The emergence of C=N groups is associated with color change from white to shades of yellow and brown, as stated in literature [14,42].

Qualitative elemental analysis of Melana indicated the presence of C, O, N, S atoms and the apparition of iodine in functionalized fibers, in addition to the above-mentioned elements (Figure 7).

**Figure 8:**
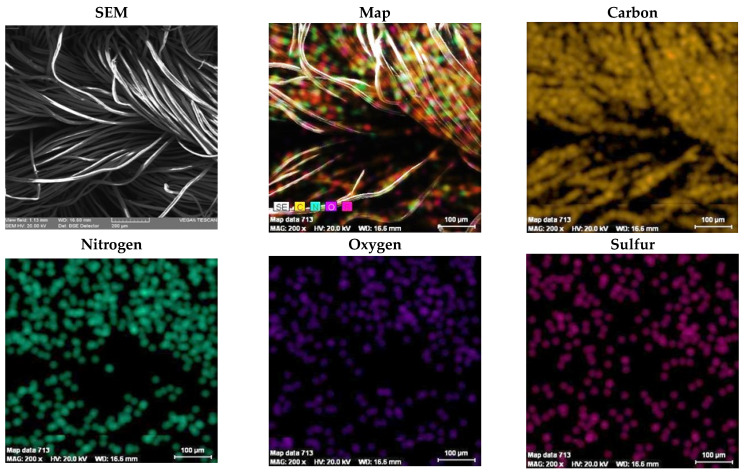
SEM image and element mapping in untreated Melana fibers.

**Figure 9:**
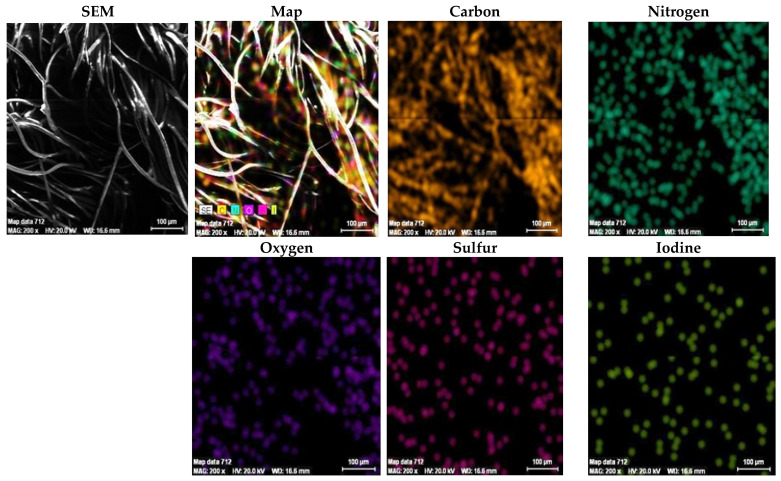
SEM image and element mapping in functionalized Melana fibers.

The presence, in Melana, of elements identified by EDX analysis (Table 4 and Figure 7) was confirmed by SEM plus map/microphotography results. Element maps recorded on Melana fibers (Figure 8 and Figure 9) showed the presence of C, N, O and S atoms. Sulphur as found in the terminal SO_3_H groups of the acrylic polymer.

The images qualitatively confirm the EDX results presented previously, namely the presence of iodine in a percentage of 1.257518% and an increase in oxygen content in functionalized Melana (15.47541% compared to 13.38738% in untreated Melana).

The element map of iodine confirmed the emergence of iodine-oxime groups during functionalization.

**Figure 10:**
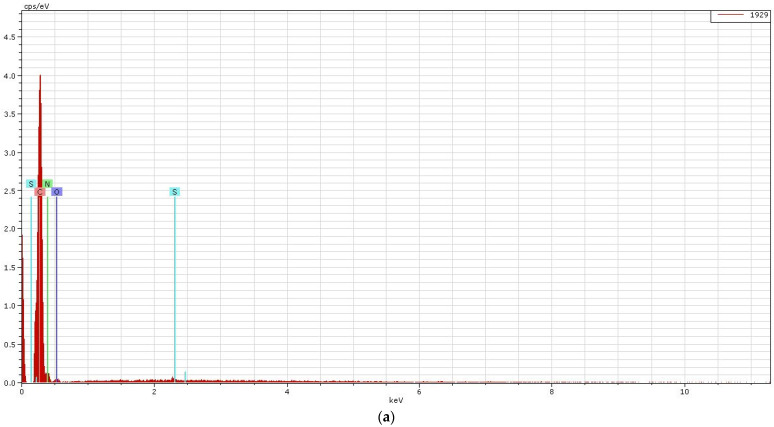

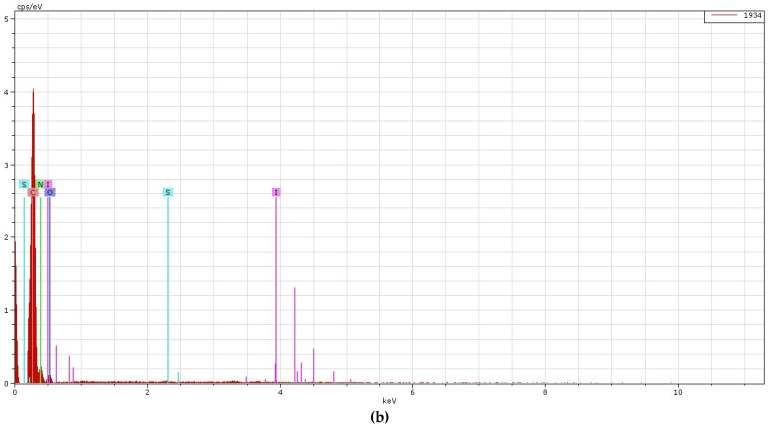
EDX spectrum of element analysis for Dralon L fibers: (**a**) untreated, and (**b**) after functionalization.

The results of the EDX analysis for the Dralon L fibers are given in Table 5 and Figure 10.

The Quantitative elemental analysis showed that the oxygen content of untreated Dralon L (11.57%) was lower than that of untreated Melana (13.38%).

For Dralon L, results of EDX analysis show that:

1. Functionalization increased the oxygen content of the polymer, from 11.57% in the treated sample, to 16.80% in the functionalized sample;
2. Compared to the untreated polymer, presence of iodine was noticed (in concentration of 2.28%); and
3. The degree of functionalization can be associated with the iodine content (2.28%) of the treated fiber.

**Figure 11:**
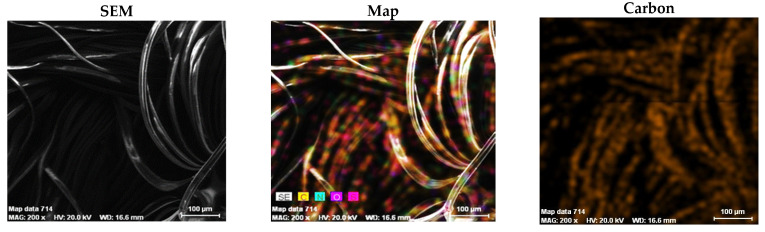

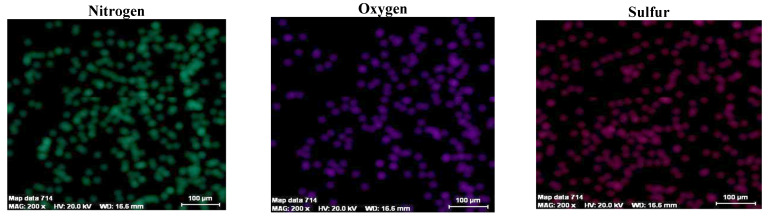
SEM image and elements mapping of untreated Dralon L fibers.

**Figure 12:**
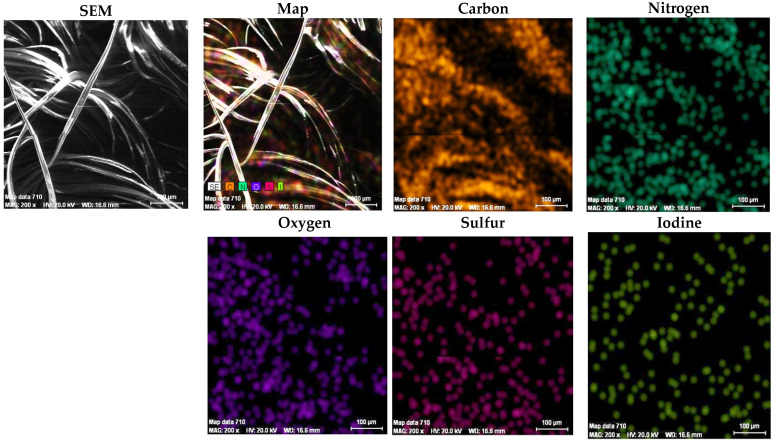
SEM image and elements mapping of functionalized Dralon L fibers.

Elements present in Dralon L’s chemical composition are visualized in the corresponding element maps from Figure 11 and Figure 12.

A correlation between EDX results and SEM images of Dralon L allows the following observations:

(a) The iodine content of functionalized Dralon L (2.28%) is higher than that of functionalized Melana (1.25%);
(b) The oxygen content of functionalized Dralon L (16.80%) is higher than that of pristine Dralon L fiber (11.57%); and
(c) The functionalization degree of Dralon L is equal to 2.28% and identical to its iodine content.

### 3.5. Thermal Resistance of Functionalized Fibers

**Figure 13:**
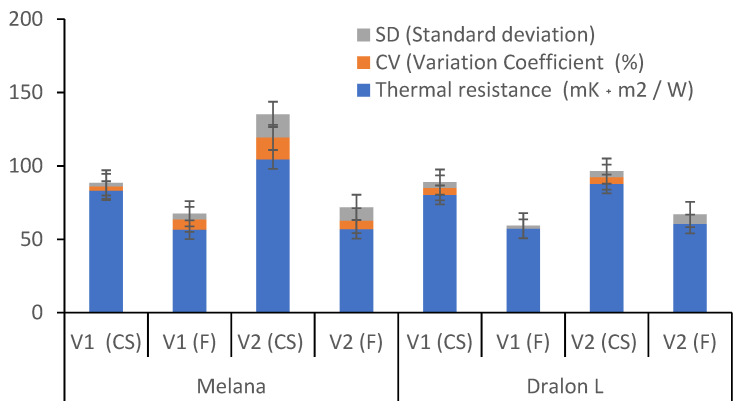
Influence of the functionalization treatment on the thermal resistance of two knitted fabric variants (V1 and V2) with acrylic fibers Melana and Dralon L. (abbreviation: CS = control sample; F = functionalization).

The values of thermal resistance before and after functionalization of PAN fibers with KOH + I_2_/KI are given in Figure 13, together with the results of statistical analysis.

In Figure 13, means of 5 determination per sample are figured, together with the results of the statistical analysis. Functionalization treatments determine the decrease of thermal resistance and increase of thermal conductivity, consequently.

Due to the specific texture of knitted fabric resulting from the curly stitch configuration, gauge value directly influenced fabric thermal resistance.

### 3.6. Results of Thermogravimetric Analysis

**Figure 14:**
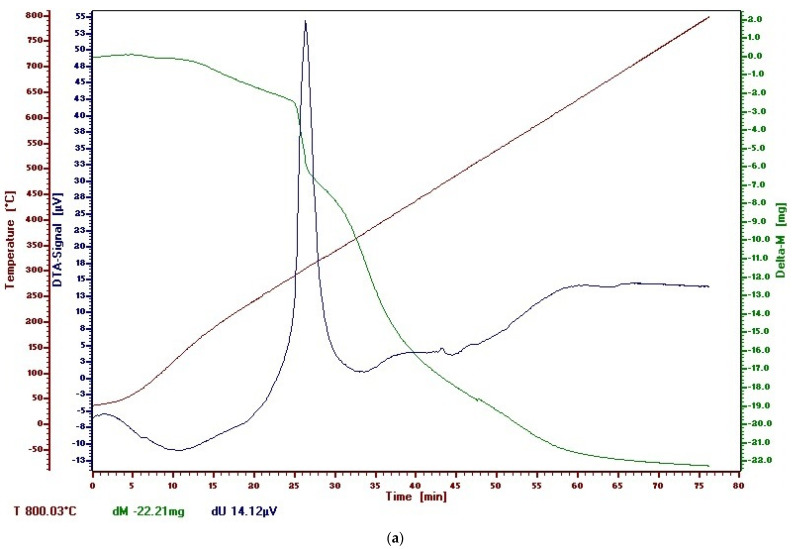

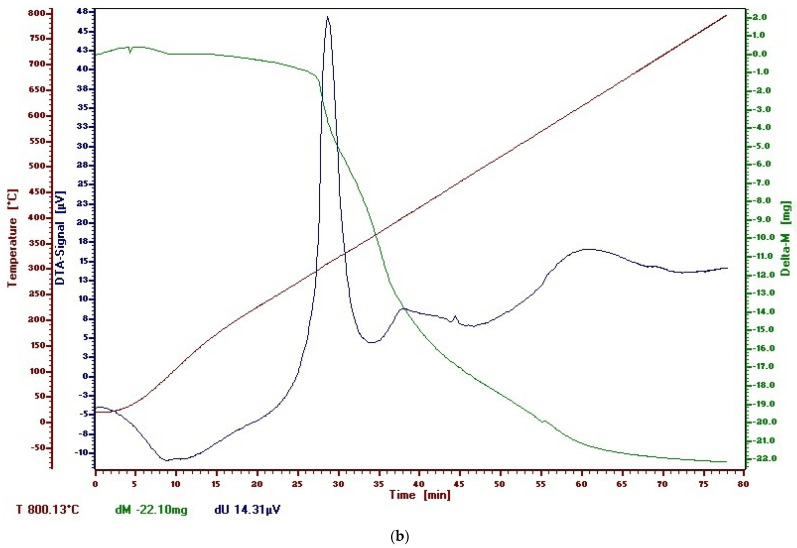

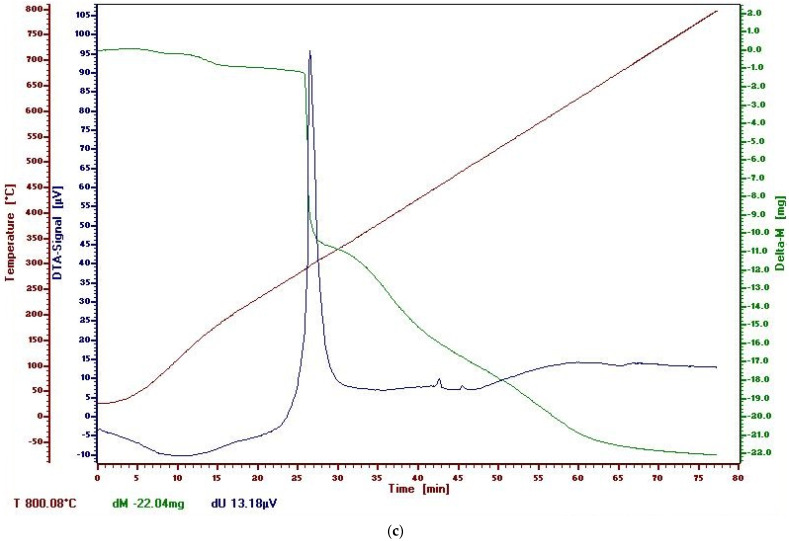

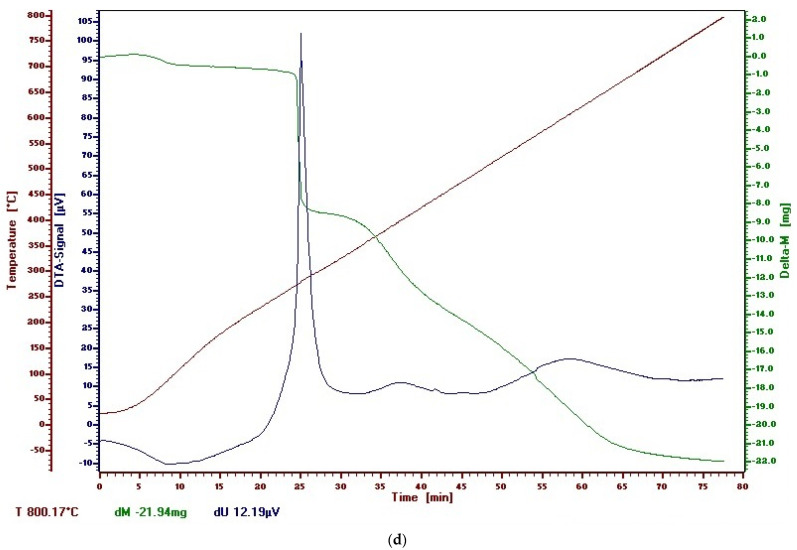
Experimental TG and DTA curves for acrylic polymers: control sample Melana (**a**), functionalized Melana (**b**), control sample Dralon L (**c**), functionalized Dralon L (**d**).

Thermogravimetric curves of untreated and functionalized PAN fibers (Figure 14) gave information regarding thermal and oxidative stability of the studied polymers.

As shown in Figure 14, both the untreated and functionalized samples underwent one-step mass changes. Thermal stability was related to the onset temperature (T_onset_), i.e., the higher to T_onset_ value, the higher thermal stability. The increase of the thermal stability of studied acrylic polymers due to functionalization is proven by the experimental values of T_onset_:

- T_onset_ of untreated Melana = 640 °C;
- T_onset_ of functionalized Melana = 680 °C;
- T_onset_ of untreated Dralon L = 670 °C; and
- T_onset_ of functionalized Dralon L = 690 °C.

This behavior can be related to the presence of iodine in the functionalized fibers.

### 3.7. Colorimetric Measurements

#### 3.7.1. Colorimetric Measurements on Samples Subjected to Severe Functionalization

**Figure 15:**
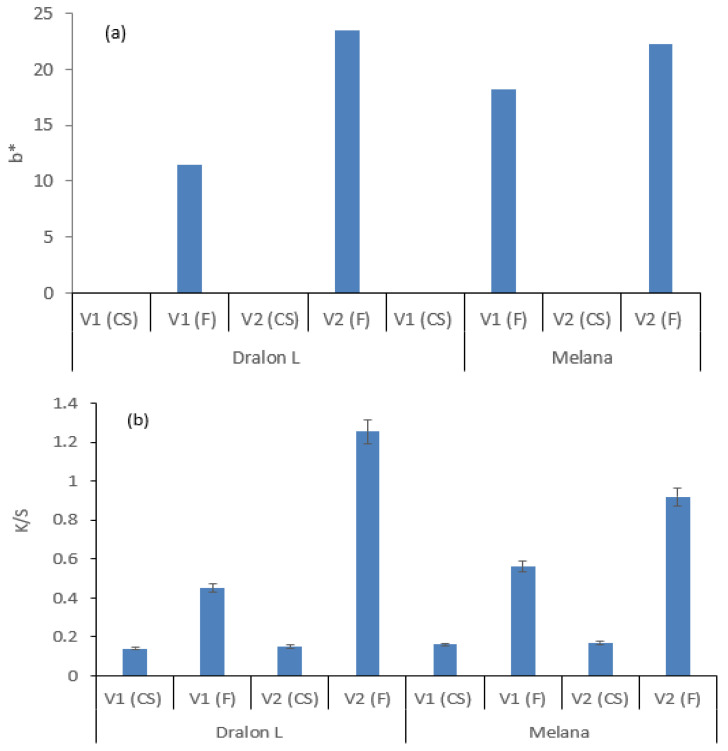
Colorimetric measurements on control sample (CS) and functionalized (F) Melana and Dralon L: (**a**) b * values; (**b**) K/S, color intensity.

Tinting of functionalized PAN was assessed by colorimetric measurements, i.e., color location on the yellow-blue axis (b *) and color strength (K/S) (Figure 15).

Severe functionalization treatments (with excess of chemicals, I_2_/KI + KOH) resulted in tinting degrees higher than standard values. Optimal treatment (controlled functionalization) parameters must be established to protect the fabric and maximize the b * and K/S values at the same time.

#### 3.7.2. Colorimetric Measurements on Samples Subjected to Controlled Functionalization

Tinting in yellow-orange shades of acrylic fabrics is obvious even with low concentrations of functionalization agents (Table 6), which is proven by:

- The increase of b * values, as compared with the standard;
- Decrease of luminosity, L *; and
- Increase of saturation, C *.

### 3.8. Influence of Functionalization Treatment upon the Color Intensity (K/S)

**Figure 16:**
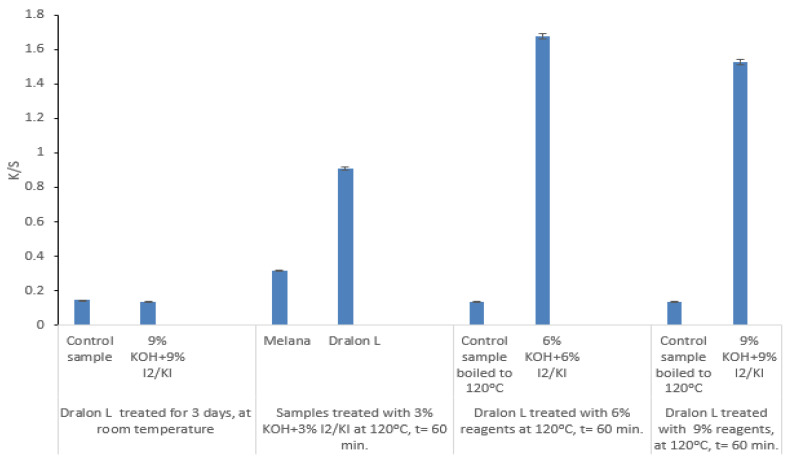
Dependence of color intensity on the functionalization treatment.

The influence of functionalization treatment upon K/S value is displayed in Figure 16. Increase of color intensity (K/S) is more noticeable in the case of controlled functionalization than in the case of severe functionalization, when chemical reagents were used in excess:

1. Color intensity depends on the functionalization degree; and
2. Color intensity depends on the working parameters and the functionalization agents:

- Treatment with I_2_/KI + KOH at room temperature did not produce any color change of the acrylic fibers: b* values increased from 0.01 for untreated Dralon L to 1.08 for Dralon L functionalized with 9% KOH + 9% I_2_/KI.
- Functionalization at 120 °C for 60 min determined a noticeable increase of b* value: 17.67 after Dralon L treatment with 3% KOH + 3% I_2_/KI and 20.7–28.47 after treatment with the same reagents but in concentrations of 6–9%.
- Functionalization resulted in fiber coloration in yellow-orange shades without using dyes.

The generation of new functional groups of iodine-oxime types (I-C=N-OH) by the conversion of a part of fraction of nitrile groups (-C≡N) determines the increase of the tinctorial capacity of acrylic fibers and creates the possibility of dyeing with acid dyes, in acid media, which is not typical for PAN fibers. The emergence of the iodine-oxime functional group was confirmed by the starch test [31,32], when the specific blue-black color that indicates the presence of I_3_^−^ in a free state (if doping takes place) did not appear.

Data from Figure 16 show that functionalization led to the generation of iodine-oxime groups and the coloration of the acrylic fibers in yellow-orange (proved by the K/S values in range 0.15 to 1.676).

**Figure 17:**
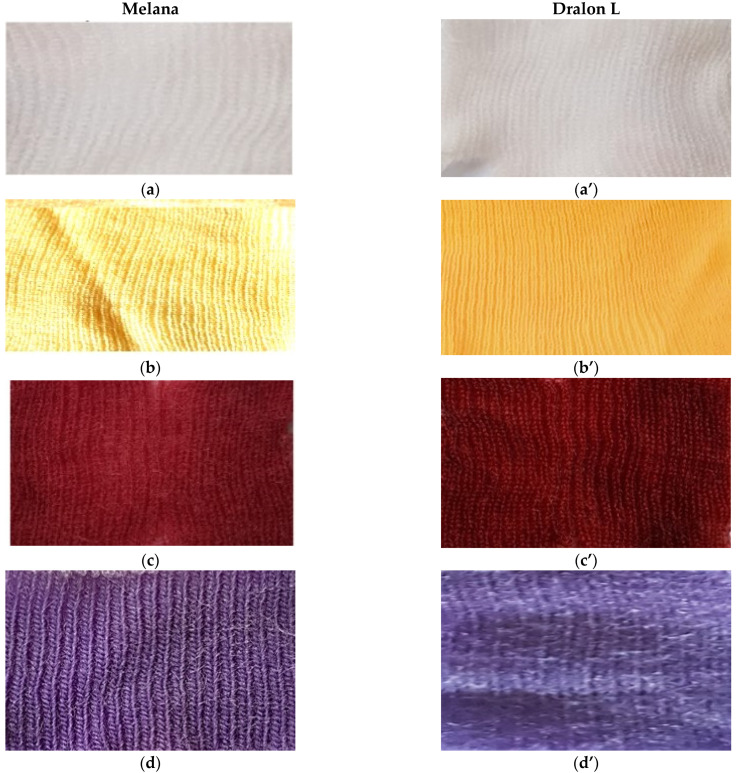
Color of acrylic fibers in different treatment stages: (**a**,**a’**) untreated, (**b**,**b’**) functionalized, and (**c**–**d’**) functionalized and dyed with acid dyes (C.I. Acid Red 57 and C.I. Acid Violet 48).

Functionalized fibers can be dyed in uniform and deep colors with non-typical dyes, such as the acid dyes C.I. Acid Red 57 (Figure 17c,d) and C.I. Acid Violet 48 (Figure 17c’,d’).

The functionalization of Melana and Dralon L fibers was confirmed by:

1. Coloration in yellow-orange, with shades depending on the treatment severity (b and b’ images in Figure 17); and
2. Affinity for non-specific/non-typical dyes and good color fastness (c,c’ and d,d’ images in Figure 17).

## 4. Conclusions

The results of this study highlight the effects of KIO_3_ (obtained in situ) on pre-oxidized and colored PAN fibers, and may open a new way to obtain the coloration of the polymer during functionalization.

Functionalization experiments on acrylic fibers Melana and Dralon L revealed:

- Coloration of PAN fibers could be a result of functionalization only, without the need to use dyestuffs;
- Coloration of treated PAN fibers depended on the functionalization degree;
- Functionalization was confirmed by the results of FTIR, SEM, Map and thermogravimetric analyses;
- Functionalization improved the fabric wearing comfort, due to the increase of fiber thermal conductibility;
- Functionalization resulted in chemical modifications of the copolymer chemical structure by conversion of some nitrile groups (C≡N) into oxime groups and the alteration of the crystalline/amorphous ratio;
- The yellow-orange color gained by functionalization exhibited fastness to repeated washings and boiling at acidic pH; and
- Functionalization of the Melana and Dralon L fibers can be easily proven by the tinctorial method, which consists in dyeing with dyes compatible with the functional group created by functionalization.

## Figures and Tables

**Table 1:** Experimental protocol for the functionalization treatments.

Functionalization Parameters	Concentration of Functionalization Agents		
	KOH [% owf] *	I_2_/KI [% owf] *	KOH + I_2_/KI [% owf] *
	**“Controlled functionalization”**		
T = 20 °C (room temperature), t = 3 days, M = 1:15	9	9	9 + 9
T = 120 °C, t = 60 min, M = 1:15	3	3	3 + 3
	6	6	6 + 6
	9	9	9 + 9
T = 120 °C, t = 30 min, M = 1:15	**“Severe functionalization”** Due to excess of chemical reagents Knit fabric variants: V1 and V2		

* % owf = percent on weight of fabric.

**Table 2:** pH and conductivity of treating liquors before and after functionalization.

Sample	Functionalization Treatment	Before Functionalization		After Functionalization	
		pH	Conductibility (μS/cm)	pH	Conductibility (μS/cm)
Dralon L	6% KOH	12.27	13,475	11.37	10,520
	6% KI	7.9	3160	7.26	3146
	6% KOH + 6% I_2_/KI	6.65	1828	4.65	2166
Melana	6% KOH	11.42	13,430	11.28	12,476
	6% KI	7.63	3166	6.31	3320
	6% KOH + 6% I_2_/KI	6.64	1753	5.25	2254

**Table 3:** FTIR adsorption bands of PAN fibers before and after functionalization with KIO_3_.

Bond	Mode	Untreated Dralon L	Dralon L	Untreated Melana	Functionalized Melana
O-H	Stretching	3627–3531	3625–3137	3637–3546	3623–3531
C-H	Stretching	2941–2873	2939–2875	2941–2869	2937–2873
C≡N	Stretching	2242	2244	2242	2242
C=O	Symmetric Stretching	1735	1735	1746	1746
C=N	Stretching	-	1629	1627	1625
CH_2_	Symmetric Deformation	1456	1456	1498–1454	1498–1454
CH_3_	Symmetric Deformation	1359	1357	1369	1371
C-O-C C-H	Stretching Wagging	1249	1226	1242	1240
C-OH	Stretching	1070	1074	1076	1076
N-O	From oxime	-	930	-	939
C–H	C–H rocking of pure PAN	839	841	863	901
for KIO_3_	-	761	-	773	
C-I Stretch		-	638	-	628

**Table 4:** Elemental composition of Melana fibers before and after functionalization.

Untreated Melana				Functionalized Melana			
Element	[wt.%]	[norm. wt.%]	[norm. at.%]	Element	[wt.%]	[norm. wt.%]	[norm. at.%]
Carbon	51.47189	51.47343	56.24074	Carbon	45.25431	45.25567	50.63058
Nitrogen	34.86316	34.86421	32.66563	Nitrogen	37.58808	37.58921	36.06172
Sulfur	0.274572	0.27458	0.112376	Sulfur	0.421683	0.421696	0.176715
Oxygen	13.38738	13.38778	10.98125	Oxygen	15.47541	15.47587	12.99782
				Iodine	1.257518	1.257556	0.133159
Sum	99.997	100	100	Sum	99.997	100	100

**Table 5:** Elemental composition of Dralon L fibers before and after functionalization.

Untreated Dralon L				Functionalized Dralon L			
Element	[wt.%]	[norm.wt.%]	[norm.at.%]	Element	[wt.%]	[norm.wt.%]	[norm.at.%]
Carbon	51.66247	51.66402	56.31075	Carbon	43.36094	43.36224	49.15906
Nitrogen	36.4963	36.49438	34.10928	Nitrogen	37.1662	37.16731	36.13237
Sulfur	0.266486	0.266494	0.108799	Sulfur	0.388544	0.388556	0.164998
Oxygen	11.57476	11.57511	9.471172	Oxygen	16.80041	16.80091	14.29883
				Iodine	2.280914	2.280983	0.244746
Sum	100	100	100	Sum	100	100	100

**Table 6:** CIELab values of acrylic fibers after controlled functionalization.

Sample Name	L *	a *	b *	C *	h	K/S
**Dralon L treatment for 3 days, at room temperature**						
Control sample	88.72	−0.74	0.01	0.74	179.42	0.1453
9% KOH	87.47	−1.22	2.48	2.75	116.13	0.1472
9% I_2_/KI	87.98	−1.66	3.57	3.94	114.95	0.1923
9% KOH + 9% I_2_/KI	86.26	−0.86	1.08	1.38	128.73	0.136
**Treatment with 3% KOH + 3% I_2_/KI at 120 °C, t = 60 min, M = 1:15**						
Melana	86.84	−0.99	13.16	13.20	94.29	0.315
Dralon L	82.29	−2.45	17.67	17.84	97.91	0.908
**Dralon L functionalization with 6% reagents at 120 °C, t = 60 min, M = 1:15**						
Control sample boiled at 120 °C (without chemicals)	88.6	−1.21	3.48	3.66	109.23	0.1351
6% KOH	80.66	1.26	33.29	33.32	87.86	2.06
6% I_2_/KI	86.8	−1.08	3.16	3.34	108.87	0.1622
6% KOH + 6% I_2_/KI	86.92	−1.56	20.73	20.79	94.31	1.676
**Dralon L functionalization with 9% reagents at 120 °C, t= 60 min, M = 1:15**						
Control sample boiled at 120 °C (without chemicals)	88.6	−1.21	3.48	3.66	109.23	0.1351
9% KOH	82.56	−0.71	24.85	24.86	91.63	1.171
9% I_2_/KI	87.02	−1.12	3.51	3.68	107.72	0.170
9% KOH + 9% I_2_/KI	81.43	−0.02	28.47	28.47	90.03	1.530
